# Supplementary material for: Mycoplasma penetrans methionyl-tRNA synthetase dimerizes via tandem N-terminal ancillary domains
Source: PLoS One. 2026 May 27;21(5):e0347747. doi: 10.1371/journal.pone.0347747 (PMC13215547; doi:10.1371/journal.pone.0347747)
Supplement: S1 File — (DOCX) [file pone.0347747.s001.docx]

**SUPPLEMENTAL FIGURES**

*Mycoplasma penetrans* methionyl-tRNA synthetase dimerizes via tandem N-terminal ancillary domains

Behrouz Ghazi Esfahani^1,2,3^, Madelynn K. Bowman^4^, Nidhi Walia^1,5^, Rebecca W. Alexander^4,^*, and M. Elizabeth Stroupe^1,^*

^1^Department of Biological Science and Institute of Molecular Biophysics, Florida State University, Tallahassee, FL, United States of America

^2^Department of Biomedical Sciences, College of Medicine, Florida State University, Tallahassee, FL, United Stated of America

^3^Current address: Department of Molecular Biosciences, Northwestern University, Evanston, IL, United States of America

^4^Department of Chemistry and Center for Molecular Signaling, Wake Forest University, Winston-Salem, NC, United States of America

^5^Current Address: Department of Biochemistry, Purdue University, West Lafayette, IN, United States of America

* Corresponding authors: Rebecca W. Alexander and M. Elizabeth Stroupe

E-mail: alexanr@wfu.edu (RWA)

E-mail: mestroupe@bio.fsu.edu (MES)

**Keywords**: Methionyl-tRNA synthetase, *Mycoplasma penetrans*, cryo-EM, class V pyridoxal phosphate-dependent aspartate aminotransferase, nucleotidyl transferase

**Running title:** *Mycoplasma penetrans* tRNA synthetase

**
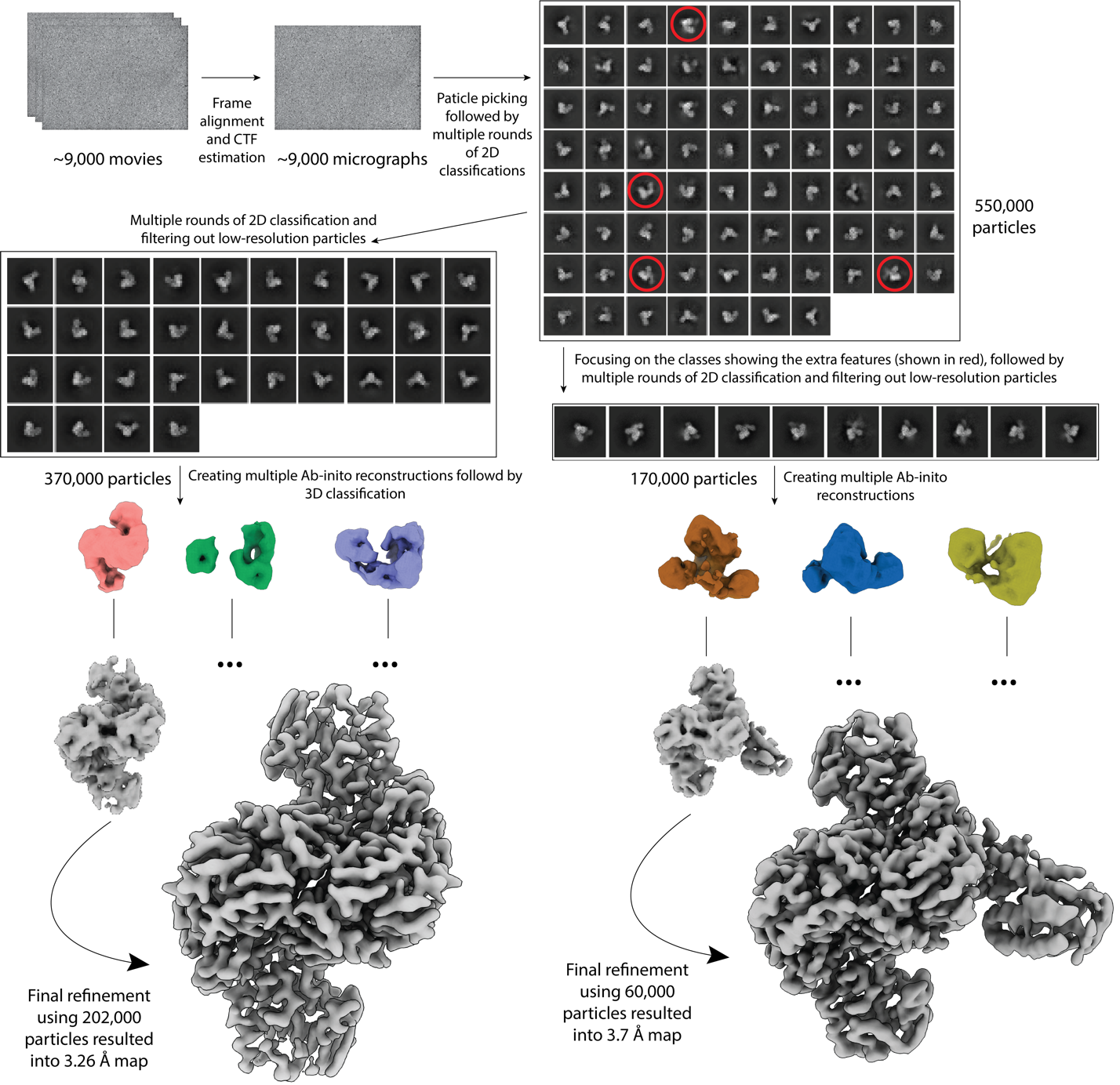
**

**Supplemental Fig 1. Image processing procedure for the MpMetRS core C2 symmetric dimer (N-terminal domain + AGAT) and the dimer including the MetRS helical subdomain.** Exemplar class averages with extra, asymmetric side density are circled in red.


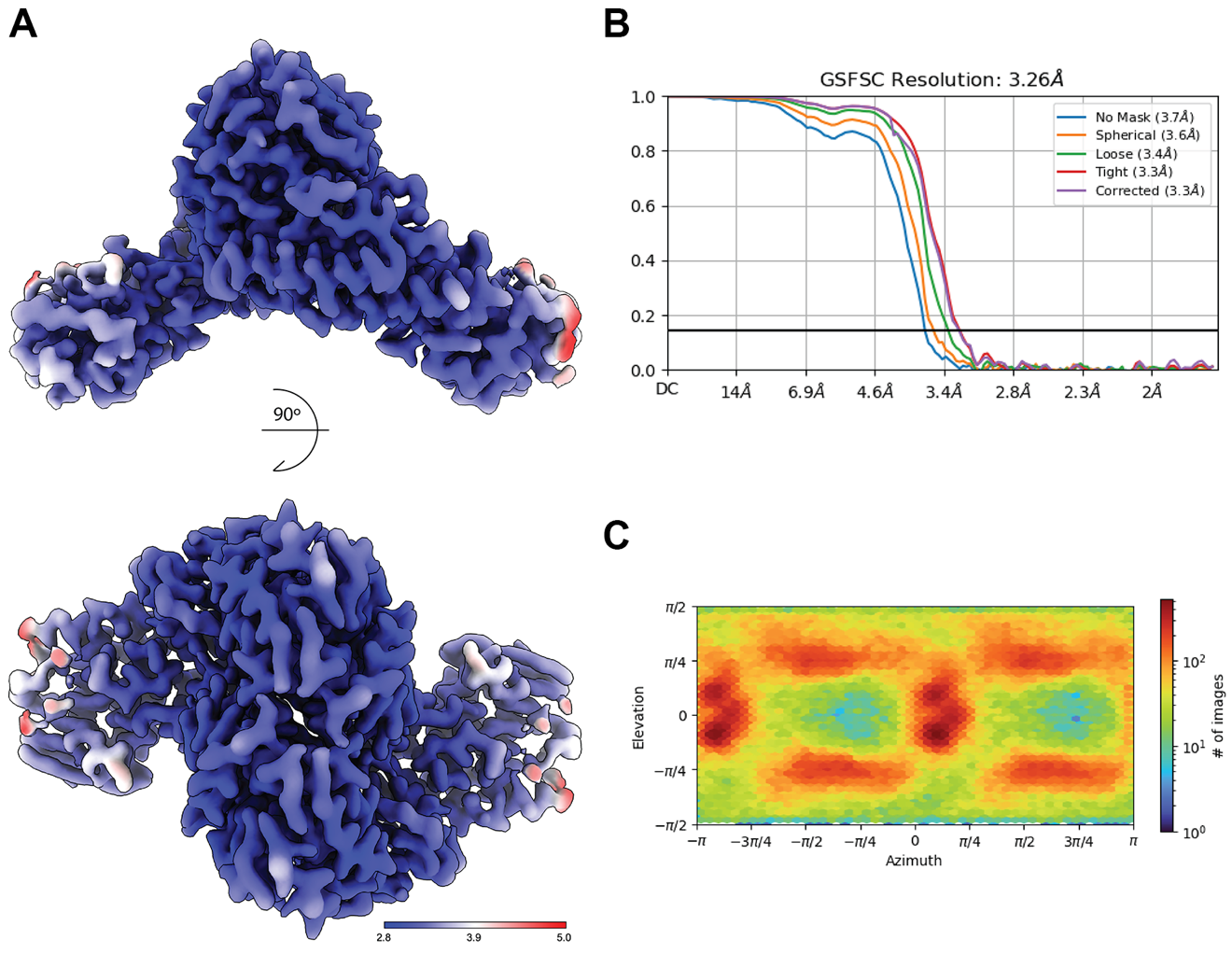


**Supplemental Figure 2: Local resolution of the core MpMetRS dimer. A.** Local resolution from 2.8 Å (blue) to 5.0 Å (red) Å mapped on the map surface. **B.** Gold Standard Fourier Shell Correlation (GSFC) analysis with various masks shows an overall resolution of 3.27 Å for the core NTD-AGAT dimer. **C.** Angular distribution of particle views that make up the core NTD-AGAT structure.

| **Data collection and processing** | **Values** |
| --- | --- |
| Voltage (kV) | 300 |
| Electron exposure (e–/Å^2^) | 60 |
| Defocus range (μm) | 0.8-2.8 |
| Pixel size (Å/pixel) | 0.86 |
| Symmetry imposed | C1 |
| Initial particle images (no.) | 550,000 |
| Final particle images (no.) | 60,000 |
| Map resolution (Å) | 3.66 |
| FSC threshold | 0.143 |
|  |  |
| **Refinement** |  |
| Map sharpening *B* factor (Å^2^) | 129.8 |
| Model composition |  |
| Non-hydrogen atoms | 10933 |
| Protein residues | 1348 |
| Average *B* factors (Å^2^) |  |
| Protein | 51.3 |
| R.M.S. deviations |  |
| Bond lengths (Å) | 0.01 |
| Bond angles (°) | 1.4 |
| Validation |  |
| MolProbity score | 2.6 |
| ClashScore | 7.8 |
| Poor rotamers (%) | 0 |
| Ramachandran plot |  |
| Favored (%) | 91.2 |
| Allowed (%) | 8.7 |
| Disallowed (%) | 0.1 |

**Supplemental Table 1: Data collection and model validation statistics**

**
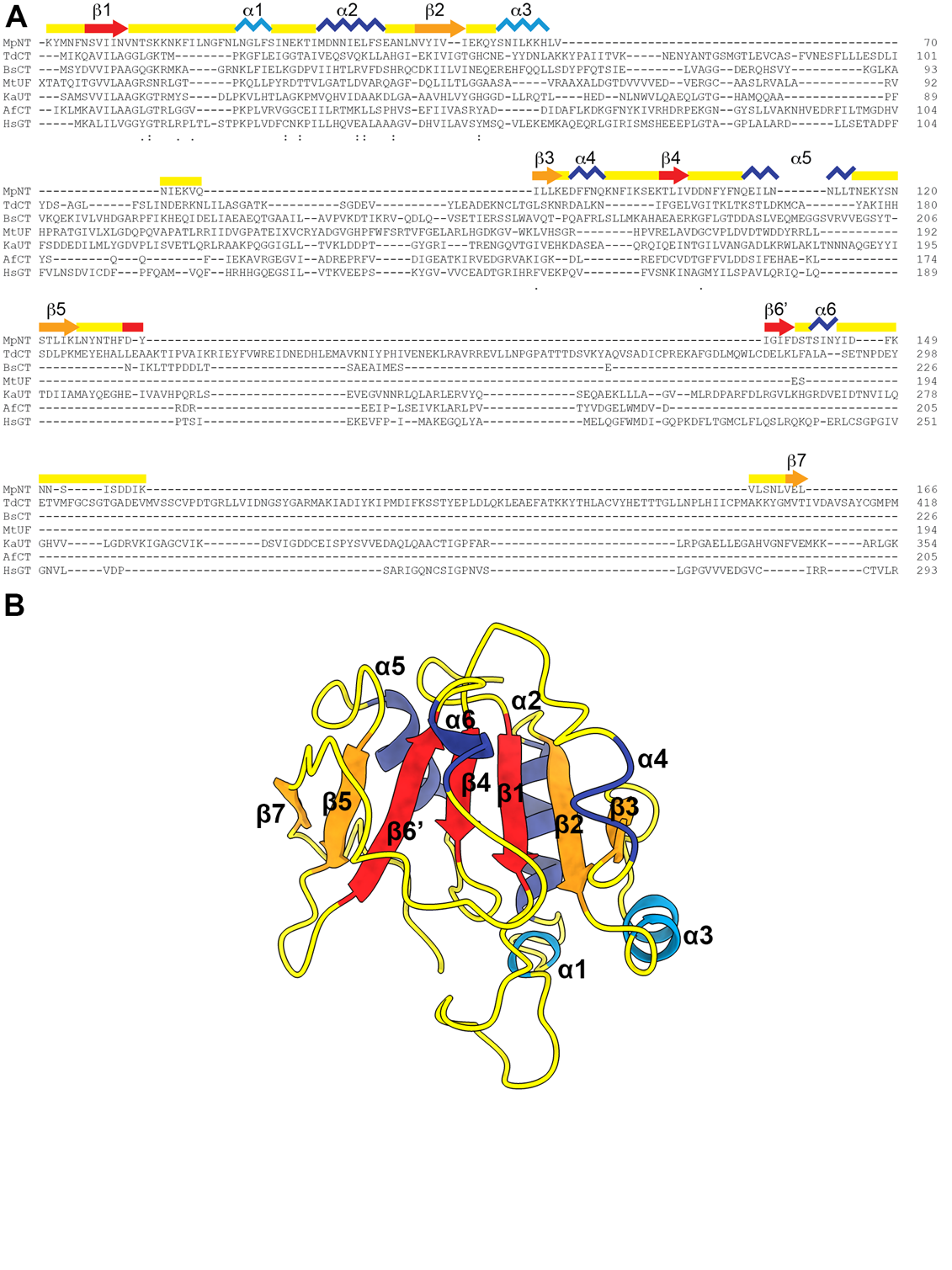
**

**Supplemental Figure 3.** **Structure-based analysis of the *MpMetRS* N-terminal domain (NTD) of unknown function. A.** Sequence alignment with non-redundant top hits from the DALI server [2] showing the secondary structure of the domain determined here (called MpNT for space) with a cytidylyltransferase from *Treponema denticola* (TdCT, PDBID 6PD1) [3]; a cytidylyltransferase from *Bacillus subtilis* (BsCT, PDBID 5HS2) [4]; a putative nucleotide transferase of unknown function from *Mycobacerium tuberculosis* (MtUF, PDBID 2WE9); a uridylyltransferase from *Klebsiella aerogenes* (KaUT, 9MH4); a cytidylyltransferase from *Archaeoglobus fulgidus* (AfCT, PDBID 2XMH) [6]; and a guanylyltransferase from *Homo sapiens* (HsGT) [7]. Secondary structure of the *MpMetRS* NTD is shown as yellow boxes (loops/turns), red arrows (NTase core β-strands) or orange arrows (peripheral β-strands, anti-parallel strands demarked as “prime”, or blue zig-zags (NTase core α-helices) or cyan zig-zags (peripheral α-helices). Sequence similarity marks homologous residues as : and similar residues as ., defined by ClustalΩ [5]. **B.** Core (red/blue) NTase structural elements with the *M. penetrans* peripheral elements (orange/cyan).

**
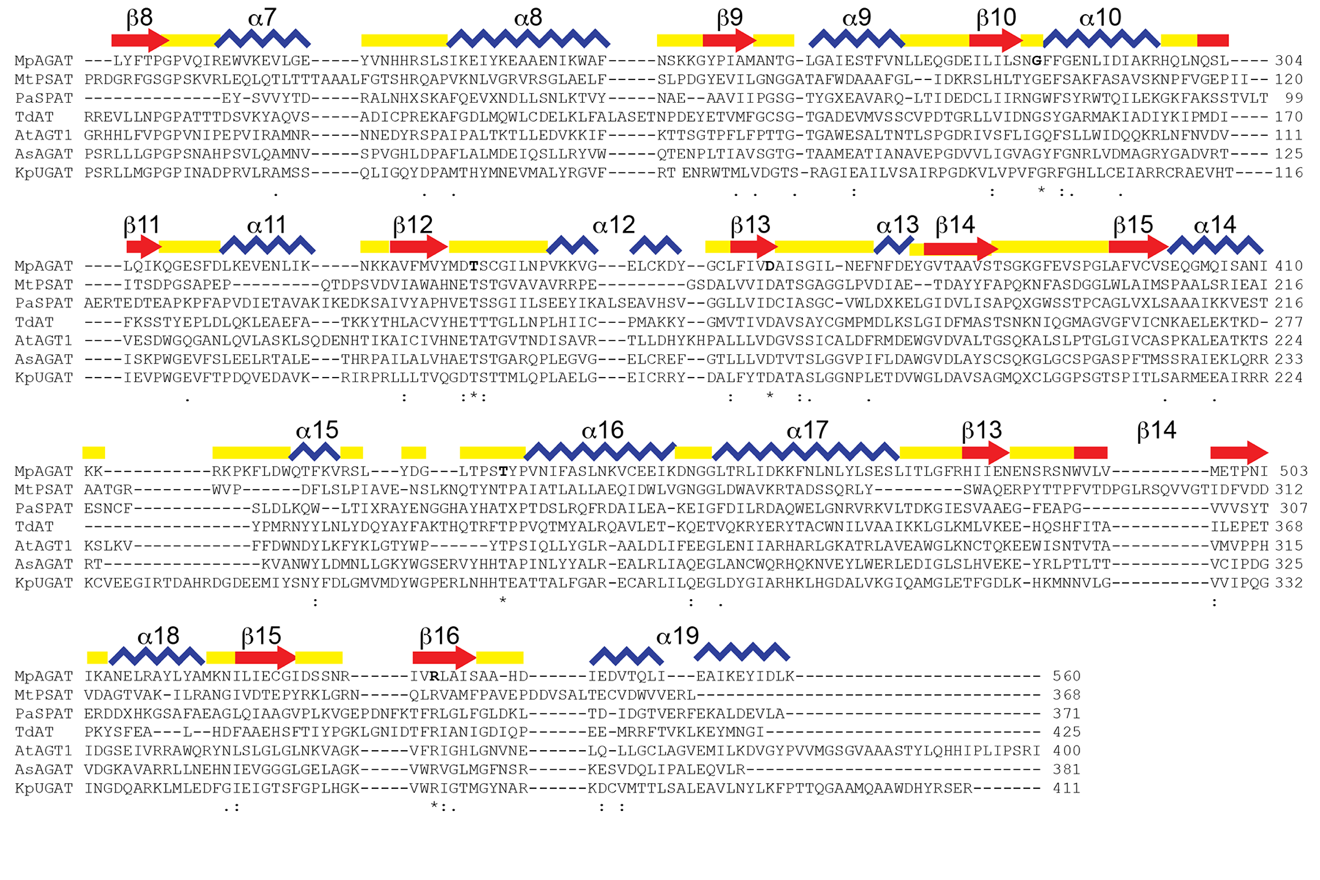
Supplemental Figure 4. Structure-based sequence analysis of the *M. penetrans* AGAT domain.** Sequence alignment with non-redundant top hits from the DALI server [2] showing the secondary structure of the domain determined here (MpAGAT) with aminotransferases that use various amine donors and acceptors, including a phosphoserine aminotransferase from *M. tuberculosis* (MtPSAT, PDBID 2FYF) [8]; a putative serine-pyruvate aminotransferase from *Psychrobacter arcticum* (PaSPAT, PDBID 3KE3); a 2-aminoethylphosphate transferase from *Treponema denticola* (TdAT, PDBID 6PD1) [3]; an alanine-glycine aminotransferase from *Arabidopsis thaliana* (AtAGT1, PDBID 6PK3) [9]; an alanine-glycine aminotransferase from *Anabaena* sp (AsAGAT, PDBID 1VJO) [10]; and a ureidoglycine aminotransferase from *Klebsiella pneumonaie* (KpUGAT, PDBID 3NNK) [11]. Secondary structure of the *M. penetrans* AGAT domain is shown as yellow boxes (loops/turns), red arrows (β strands, anti-parallel demarked with as “prime”, or blue zig-zags (α-helices)). Sequence similarity marks identical residues as * (bold in the MpAGAT sequence), conserved residues as : and similar residues as ., defined by ClustalΩ [5].


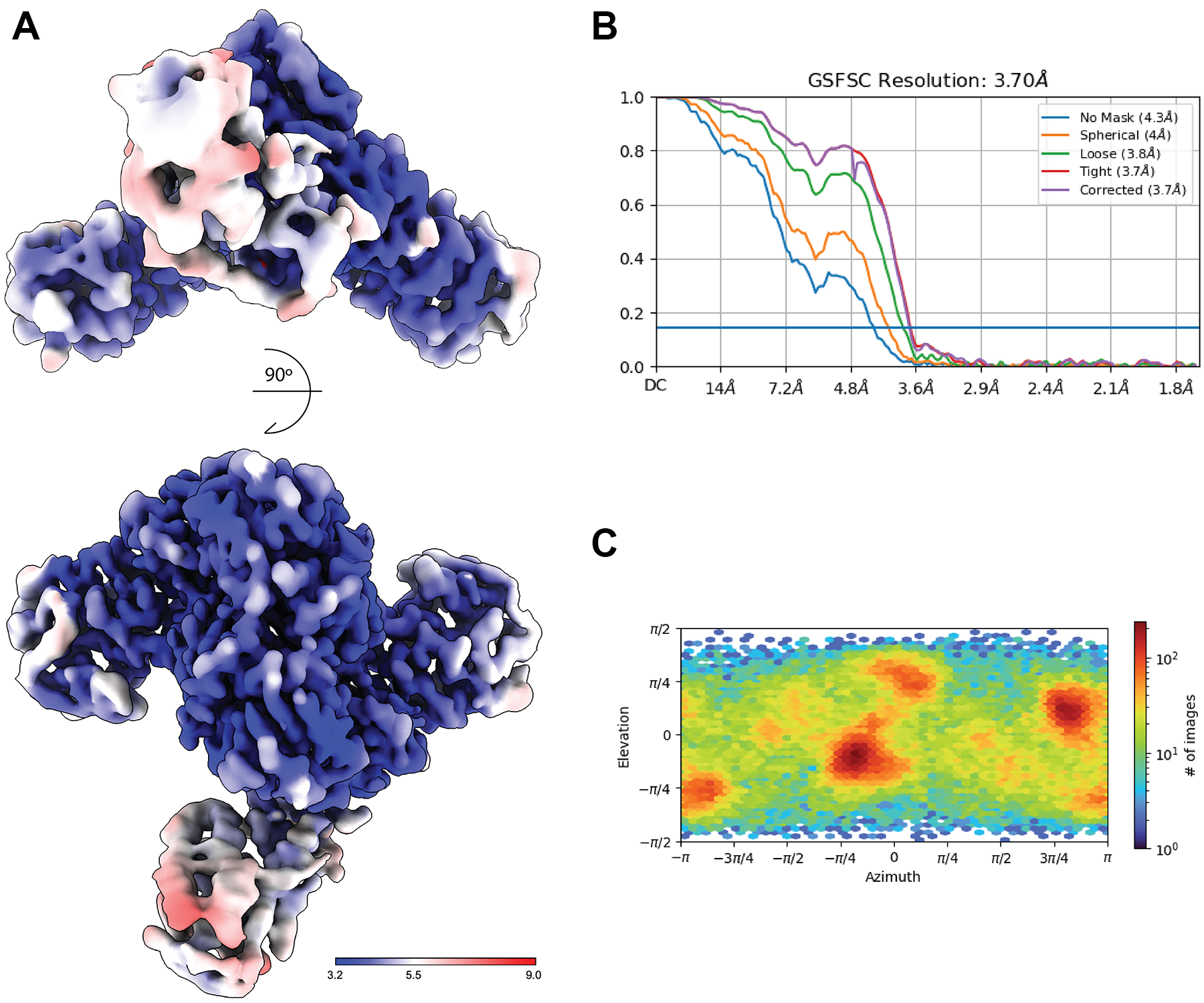


**Supplemental Figure 5: Local resolution of the NDT-AGAT-helical subdomain of the MetRS domain. A.** Local resolution from 3.2 Å (blue) to 9.0 Å (red) mapped on the map surface. **B.** Gold Standard Fourier Shell Correlation (GSFC) analysis with various masks shows an overall resolution of 3.66 Å for the three visible domains. **C.** Angular distribution of particle views that make up the core NTD-AGAT structure.


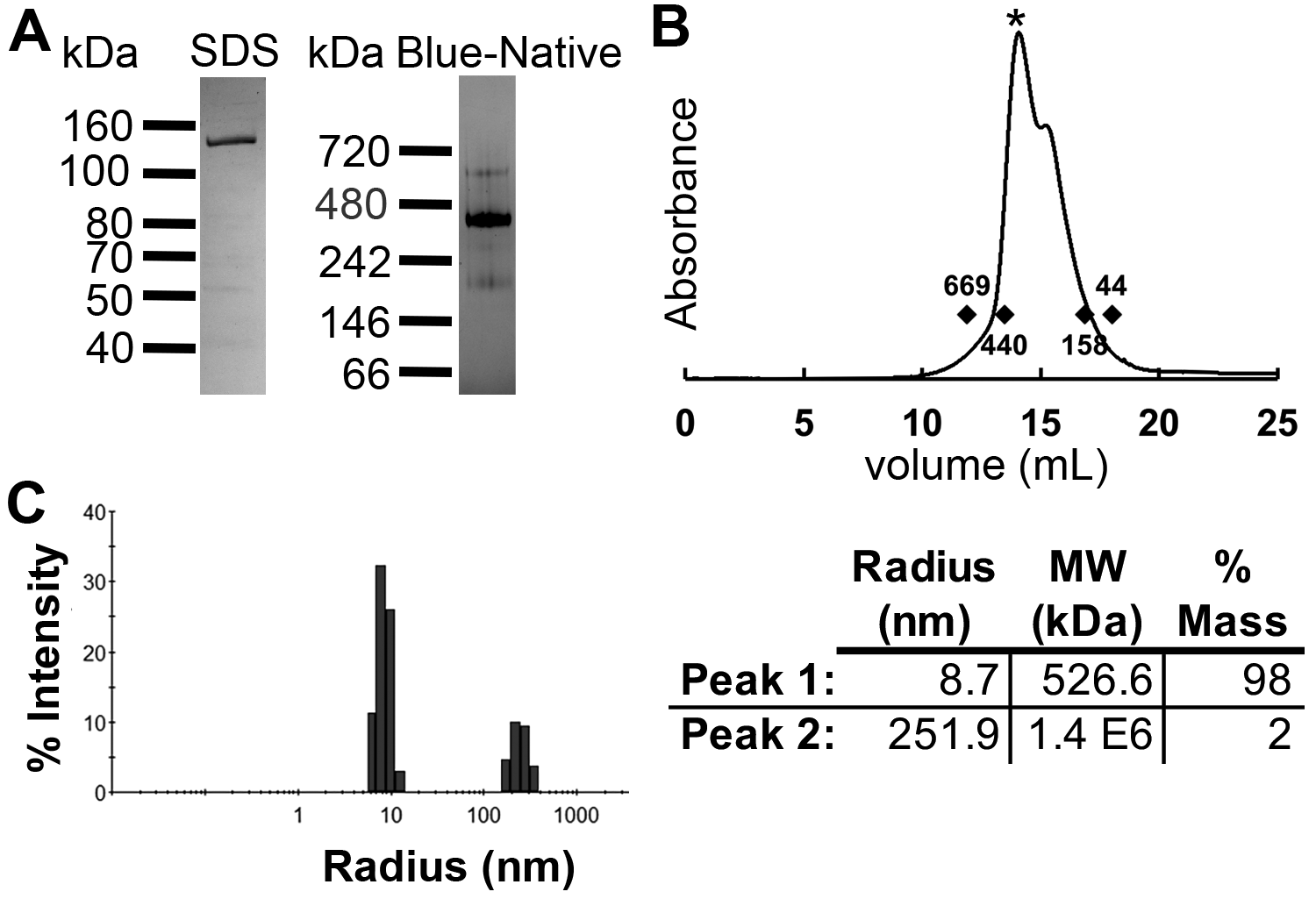


**Figure S1.** Structure-based analysis of the *MpMetRS* N-terminal domain of unknown function. **A.** Amino acid alignment with non-redundant top hits from the DALI server [2] showing the secondary structure of the domain determined here (called MpNT here for space) with a cytidylyltransferase from *Treponema denticola* (TdCT); a cytidylyltransferase from *Bacillus subtilus* (BsCT); a putative nucleotide transferase of unknown function from *Mycobacerium tuberculosis* (MtUF); a uridylyltransferase from *Klebsiella aerogenes* (KaUT); a cytidylyltransferase from *Archaeoglobus fulgidus* (AfCT); and a guanyltransferase from *Homo sapien* (HsGT). Secondary structure of the *MpMetRS* N-terminal domain of unknown function is shown as yellow boxes (loops/turns), red arrows (β strands, anti-parallel demarked with as “prime”, or blue zig-zags (α-helices)). Sequence similarity shows conserved residues as : and similar residues as ., defined by ClustalΩ [5]. **B.** Structure-based similarity of the seven folds, showing a highly conserved core (blue) with varying peripheral helices and loops, as well as variability in the central β-sheet. C. Table summarizing the reference PDBs for each organism/sucleotide specificity, and core similarity.

**Supplemental Figure 6. Molecular Weight Analysis of MpMetRS. A.** PAGE (SDS or Blue Native) analysis. **B.** SEC analysis by a Superose6 column (Cytiva, Marlborough, MA, USA) with high molecular weight markers (ThermoScientific, Waltham, MA, USA) as diamonds. The peak that was used to prepare cryo-EM samples is marked with an asterisk (*). **C.** Dynamic Light Scattering analysis of 5 mg/mL MpMetRS shows a hydrodynamic radius of about 9 nm.

| **Protein/domain** | **theoretical MW (kDa)** | **dimer MW (kDa)** |
| --- | --- | --- |
| MpMetRS | 126.3 | 252.6 |
| NTD | 23.7 |  |
| AGAT | 40.5 | 81 |
| NTD-AGAT | 64.2 | 128.4 |
| MetRS | 62.1 |  |
| MetRS-Nterm | 2.4 |  |
| MetRS-helical | 59.7 |  |

**Supplemental Table 2. Molecular weights (MWs) of MpMetRS domains**


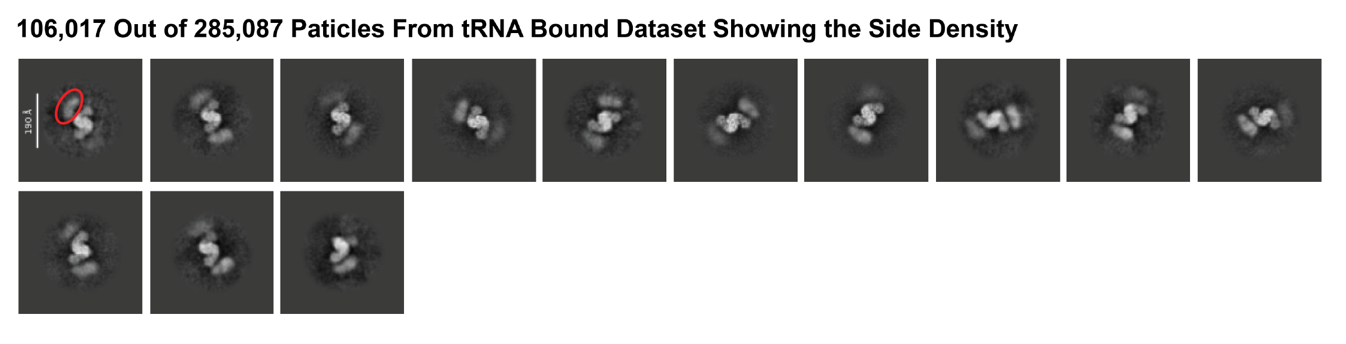


**Supplemental Figure 7:** A subset of particles from the tRNA bound dataset showed peripheral side density, for example marked by a red oval, but that showed no density by 3D reconstruction.

**References:**

1. Pettersen EF, Goddard TD, Huang CC, Meng EC, Couch GS, Croll TI, et al. UCSF ChimeraX: Structure Visualization for Researchers, Educators, and Developers. Protein Sci. 2020. Epub 2020/09/03. doi: 10.1002/pro.3943. PubMed PMID: 32881101.

2. Holm L, Laiho A, Törönen P, Salgado M. DALI shines a light on remote homologs: One hundred discoveries. Protein Sci. 2023;32(1):e4519. doi: 10.1002/pro.4519. PubMed PMID: 36419248; PubMed Central PMCID: PMCPMC9793968.

3. Rice K, Batul K, Whiteside J, Kelso J, Papinski M, Schmidt E, et al. The predominance of nucleotidyl activation in bacterial phosphonate biosynthesis. Nat Commun. 2019;10(1):3698. Epub 20190816. doi: 10.1038/s41467-019-11627-6. PubMed PMID: 31420548; PubMed Central PMCID: PMCPMC6697681.

4. Jin Y, Liu Z, Li Y, Liu W, Tao Y, Wang G. A structural and functional study on the 2-C-methyl-d-erythritol-4-phosphate cytidyltransferase (IspD) from Bacillus subtilis. Sci Rep. 2016;6:36379. Epub 20161108. doi: 10.1038/srep36379. PubMed PMID: 27821871; PubMed Central PMCID: PMCPMC5099578.

5. Sievers F, Higgins DG. Clustal Omega for making accurate alignments of many protein sequences. Protein Sci. 2018;27(1):135-45. Epub 2017/10/30. doi: 10.1002/pro.3290. PubMed PMID: 28884485; PubMed Central PMCS4ID: PMCPMC5734385.

6. Brito JA, Borges N, Vonrhein C, Santos H, Archer M. Crystal structure of Archaeoglobus fulgidus CTP:inositol-1-phosphate cytidylyltransferase, a key enzyme for di-myo-inositol-phosphate synthesis in (hyper)thermophiles. J Bacteriol. 2011;193(9):2177-85. Epub 20110304. doi: 10.1128/JB.01543-10. PubMed PMID: 21378188; PubMed Central PMCID: PMCPMC3133074.

7. Zheng L, Liu Z, Wang Y, Yang F, Wang J, Huang W, et al. Cryo-EM structures of human GMPPA-GMPPB complex reveal how cells maintain GDP-mannose homeostasis. Nat Struct Mol Biol. 2021;28(5):1-12. Epub 20210513. doi: 10.1038/s41594-021-00591-9. PubMed PMID: 33986552.

8. Coulibaly F, Lassalle E, Baker HM, Baker EN. Structure of phosphoserine aminotransferase from Mycobacterium tuberculosis. Acta Crystallogr D Biol Crystallogr. 2012;68(Pt 5):553-63. Epub 20120417. doi: 10.1107/S0907444912004829. PubMed PMID: 22525753.

9. Liepman AH, Vijayalakshmi J, Peisach D, Hulsebus B, Olsen LJ, Saper MA. Crystal Structure Of Photorespiratory Alanine:Glyoxylate Aminotransferase 1 (AGT1) From. Front Plant Sci. 2019;10:1229. Epub 20191011. doi: 10.3389/fpls.2019.01229. PubMed PMID: 31681359; PubMed Central PMCID: PMCPMC6797613.

10. Han GW, Schwarzenbacher R, Page R, Jaroszewski L, Abdubek P, Ambing E, et al. Crystal structure of an alanine-glyoxylate aminotransferase from Anabaena sp. at 1.70 A resolution reveals a noncovalently linked PLP cofactor. Proteins. 2005;58(4):971-5. doi: 10.1002/prot.20360. PubMed PMID: 15657930.

11. French JB, Cen Y, Sauve AA, Ealick SE. High-resolution crystal structures of Streptococcus pneumoniae nicotinamidase with trapped intermediates provide insights into the catalytic mechanism and inhibition by aldehydes. Biochemistry. 2010;49(40):8803-12. Epub 20100920. doi: 10.1021/bi1012436. PubMed PMID: 20853856; PubMed Central PMCID: PMCPMC3006156.
