# Supplementary material for: Mycoplasma penetrans methionyl-tRNA synthetase dimerizes via tandem N-terminal ancillary domains
Source: PLoS One. 2026 May 27;21(5):e0347747. doi: 10.1371/journal.pone.0347747 (PMC13215547; doi:10.1371/journal.pone.0347747)
Supplement: Attachment — Submitted filename: PlosOne_RtR.docx [file pone.0347747.s003.docx]

**Journal Requirements:**

When submitting your revision, we need you to address these additional requirements.

1. Please ensure that your manuscript meets PLOS ONE's style requirements, including those for file naming. The PLOS ONE style templates can be found at

https://journals.plos.org/plosone/s/file?id=wjVg/PLOSOne_formatting_sample_main_body.pdf and

<https://journals.plos.org/plosone/s/file?id=ba62/PLOSOne_formatting_sample_title_authors_affiliations.pdf>

The title page has been formatted according to the style requirements.

2. Please note that PLOS One has specific guidelines on code sharing for submissions in which author-generated code underpins the findings in the manuscript. In these cases, we expect all author-generated code to be made available without restrictions upon publication of the work. Please review our guidelines at https://journals.plos.org/plosone/s/materials-and-software-sharing#loc-sharing-code and ensure that your code is shared in a way that follows best practice and facilitates reproducibility and reuse.

No unique code was used in this study.

3. Thank you for stating the following financial disclosure:

“We thank Dr. Christopher Stroupe and Jen Kennedy for helpful discussions. Florida State University supports cryo-EM in the Biological Imaging Resource Center, which houses the following equipment used in this study: a Gatan Solaris Plasma Cleaner (NIH grant S10 RR024564), a Hitachi HT7800 (NSF grant MRI2017869 to M.E.S.), a ThermoFisher Vitrobot Mark 14 IV (NIH grant S10 RR024564), an SPI chameleon® plunging system (NIH grant R24 GM145964),a ThermoFisher Titan Krios (NIH grant S10 RR025080), and a DE Apollo direct electron detector (NIH grant R35 GM139616). This work was further supported by National Science Foundation grants MCB1856502 and CHE1904612 to M.E.S. “

Please state what role the funders took in the study. If the funders had no role, please state: "The funders had no role in study design, data collection and analysis, decision to publish, or preparation of the manuscript."

If this statement is not correct you must amend it as needed.

Please include this amended Role of Funder statement in your cover letter; we will change the online submission form on your behalf.

The statement is correct, and we have included it in our revised cover letter.

4. Thank you for stating the following in the Acknowledgments Section of your manuscript:
“We thank Dr. Christopher Stroupe and Jen Kennedy for helpful discussions. Florida State University supports cryo-EM in the Biological Imaging Resource Center, which houses the following equipment used in this study: a Gatan Solaris Plasma Cleaner (NIH grant S10 RR024564), a Hitachi HT7800 (NSF grant MRI2017869 to M.E.S.), a ThermoFisher Vitrobot Mark 14 IV (NIH grant S10 RR024564), an SPI chameleon® plunging system (NIH grant R24 GM145964),a ThermoFisher Titan Krios (NIH grant S10 RR025080), and a DE Apollo direct electron detector (NIH grant R35 GM139616). This work was further supported by National Science Foundation grants MCB1856502 and CHE1904612 to M.E.S. “

We note that you have provided funding information that is not currently declared in your Funding Statement. However, funding information should not appear in the Acknowledgments section or other areas of your manuscript. We will only publish funding information present in the Funding Statement section of the online submission form.

Please remove any funding-related text from the manuscript and let us know how you would like to update your Funding Statement. Currently, your Funding Statement reads as follows:
“The author(s) received no specific funding for this work”

Please include your amended statements within your cover letter; we will change the online submission form on your behalf.

We have updated the funding statement, as presented in the cover letter.

5. We note that the grant information you provided in the ‘Funding Information’ and ‘Financial Disclosure’ sections do not match.

When you resubmit, please ensure that you provide the correct grant numbers for the awards you received for your study in the ‘Funding Information’ section.

We have moved this information to the Funding Information section.

6. If the reviewer comments include a recommendation to cite specific previously published works, please review and evaluate these publications to determine whether they are relevant and should be cited. There is no requirement to cite these works unless the editor has indicated otherwise.

Noted. Thank you.

[Note: HTML markup is below. Please do not edit.]

Reviewers' comments:

Reviewer's Responses to Questions

**Comments to the Author**

1. Is the manuscript technically sound, and do the data support the conclusions?

The manuscript must describe a technically sound piece of scientific research with data that supports the conclusions. Experiments must have been conducted rigorously, with appropriate controls, replication, and sample sizes. The conclusions must be drawn appropriately based on the data presented.

Reviewer #1: Partly

Reviewer #2: Yes

Reviewer #3: Partly

Reviewer #4: Partly

2. Has the statistical analysis been performed appropriately and rigorously?

Reviewer #1: I Don't Know

Reviewer #2: Yes

Reviewer #3: Yes

Reviewer #4: N/A

3. Have the authors made all data underlying the findings in their manuscript fully available?

The [PLOS Data policy](http://www.plosone.org/static/policies.action#sharing) requires authors to make all data underlying the findings described in their manuscript fully available without restriction, with rare exception (please refer to the Data Availability Statement in the manuscript PDF file). The data should be provided as part of the manuscript or its supporting information, or deposited to a public repository. For example, in addition to summary statistics, the data points behind means, medians and variance measures should be available. If there are restrictions on publicly sharing data—e.g. participant privacy or use of data from a third party—those must be specified.

Reviewer #1: Yes

Reviewer #2: Yes

Reviewer #3: Yes

Reviewer #4: Yes

4. Is the manuscript presented in an intelligible fashion and written in standard English?

PLOS ONE does not copyedit accepted manuscripts, so the language in submitted articles must be clear, correct, and unambiguous. Any typographical or grammatical errors should be corrected at revision, so please note any specific errors here.

Reviewer #1: Yes

Reviewer #2: Yes

Reviewer #3: Yes

Reviewer #4: Yes

5. Review Comments to the Author

Please use the space provided to explain your answers to the questions above. You may also include additional comments for the author, including concerns about dual publication, research ethics, or publication ethics. (Please upload your review as an attachment if it exceeds 20,000 characters)

**Reviewer #1: Major revision**

1. In the background, authors can state more about the progress of MetRS structure, and then introduce the aim of this study.

We have included this information in new paragraph 5, Introduction:

“AARSs from diverse taxa have been biochemically dissected to explain their ancient and essential role in faithfully translating the genetic code, so much is known about the induced fit mechanism that allows the enzyme to discriminate amongst similar amino acid functional groups to pair them with a three-nucleotide anticodon. Nevertheless, high-resolution structure determination techniques rely on specimen homogeneity, so many of the existing AARS structures have missing features that do not fully define the structural basis for the AARS mechanism. For example, tRNAs that are aminoacylated by class I sub-family AARSs necessarily undergo a “hairpin” rearrangement of the 3’-terminal acceptor end to approach the activated amino acid in the AARS catalytic site. Few high-resolution structures of such hairpinned tRNAs bound to their cognate AARSs are available. The modular nature of AARSs contributes to efficient catalysis and a wide array of novel functions but a complete model of the structural basis for the biochemical data remains elusive. In the case of MpMetRS, ambiguity in sequence homology of the N-terminal domain of unknown function necessitated further experimental analysis.”

2. In fact, the Cryo-EM sample preparation is the main part of this study, which determined the final results, and it can describe the protocol in details.

We have added the following details to the Materials and Methods:

“Further refinement of the apo-MpMetRS dataset by template-free 2D classification identified 202,593 particles with clear 2-fold symmetry that were used for initial model building. Non-uniform 3D refinement with imposed C2 symmetry resulted in a 3.27 Å-resolution map. The map showed no evidence of the MetRS catalytic domain. Closer inspection of the 2D classes revealed some classes with blurred features that did not follow the 2-fold symmetry that dominated the initial classification. To explore the possibility that this density corresponded to the missing MetRS catalytic domain, this limited subset of particles (60,000) was used to refine a non-uniform, asymmetric map to 3.66 Å resolution. All maps were sharpened with deepEMhancer for enhanced visualization .”

3. It should compare current results with others, and show the unique of MetRS in structure and explain the potential impacts of Mycoplasma penetrans.

We have expanded our analysis to compare in detail the N-terminal domains (the domain of unknown function) with homology to other structural homologs.

Minor revision

1. The resolution of figure 1 can be improved.

We have regenerated all figures as tif files to a resolution of 300 d.p.i. with a width of 5.2 inches.

2. Figure 2 and 3 can integrate into one.

We have reorganized the figures to enhance the readability and flow of the manuscript.

**Reviewer #2: Manuscript ID: PONE-D-25-61422**

Recommendation: Major Revision

Reviewer: Morufu Olalekan Raimi, PhD

Affiliation: Niger Delta Institute for Emerging and Re-emerging Infectious Diseases, Federal University, Otuoke, Bayelsa State

Review Date: 4-December, 2024

Dear Editors and Authors,

Thank you for the opportunity to review the manuscript titled “Mycoplasma penetrans methionyl tRNA synthetase is an asymmetric dimer fused to N-terminal ancillary domains.” The study presents a structural investigation of a unique chimeric MetRS enzyme from M. penetrans, employing cryo-EM to elucidate its domain architecture and conformational dynamics. The work addresses an interesting evolutionary and functional question in the context of streamlined genomes and domain fusion events. While the structural data and overall premise are promising, several significant concerns must be addressed before the manuscript can be considered for publication.

i. Title and Abstract

• Title: The title is clear and accurately reflects the content.

• Abstract: The abstract succinctly summarizes the study but could be strengthened by explicitly stating the resolution of the cryo-EM map and the functional implications of the observed asymmetry. Currently, the abstract reads as descriptive; it should better highlight the novelty e.g., the first structure of a triple-domain MetRS fusion and its implications for substrate channeling in a minimal genome organism.

We added the following to the abstract:

“We used cryo-EM to analyze the structure of the MpMetRS gene product to show that it is the product of three distinct enzyme domains: an N-terminal domain (NTD) of unknown function, a dimeric alanine-glyoxylate aminotransferase, and a MetRS. Only the first two N-terminal domains show two-fold symmetry, which were resolved to 3.27 Å resolution and the MetRS domain is only partially resolved to 3.66 Å resolution. Modelling the full structure shows that a conformational change must occur to accommodate a tRNA-bound MetRS domain. Further rearrangement of the catalytic domains would also be necessary to bring the active sites adjacent to one another if this unique assembly of catalytic domains functions to channel substrates to MetRS.”

ii. Introduction

The introduction adequately contextualizes M. penetrans and the peculiarity of its metS gene. However, it lacks a clear thematic bridge between domain fusion, structural asymmetry, and functional adaptation in parasitic bacteria.

• Author should expand on why structural asymmetry might be evolutionarily advantageous in this context.

Additionally, briefly introduce the concept of substrate channeling in fused enzymes to frame the study’s hypothesis more explicitly.

We thank the reviewer for these helpful suggestions. As we do not have direct observations of a substrate channeling complex, we opted to enhance our discussion of the variety of AARS fusions in the literature as follows to new paragraph 5, Introduction:

“AARSs from diverse taxa have been biochemically dissected to explain their ancient and essential role in faithfully translating the genetic code. Many AARSs exhibit induced fit, both to select their cognate tRNAs based on nucleotide identities in the three-nucleotide anticodon and the acceptor stem and to discriminate amongst similar amino acid functional groups. Nevertheless, high-resolution structure determination techniques rely on specimen homogeneity, so many of the existing AARS structures have missing features that do not fully define the structural basis for the AARS mechanism. For example, tRNAs that are aminoacylated by class I sub-family AARSs necessarily undergo a “hairpin” rearrangement of the 3’-terminal acceptor end to approach the activated amino acid in the AARS catalytic site. Few high-resolution structures of such hairpinned tRNAs bound to their cognate AARSs are available. The modular nature of AARSs contributes to efficient catalysis and a wide array of novel functions but a complete model of the structural basis for the biochemical data remains elusive. In the case of MpMetRS, ambiguity in sequence homology of the N-terminal domain of unknown function necessitated further experimental analysis.”

iii. Materials and Methods

This part is generally well-described but requires clarification in several areas:

1. Sample Preparation:

o The concentration of MpMetRS used for cryo-EM (0.04 mg/mL) is extremely low for typical cryo-EM studies. Justify this choice and discuss potential implications for particle distribution and ice quality.

We extensively screened protein concentrations and found that the protein aggregates (or polymerizes, see below) at high concentrations. The graphene substrate adheres to the protein, thus concentrating it in the ice before blotting and plunge freezing. To clarify, we added the following to Materials and Methods/Cryo-EM sample preparation:

“MpMetRS adheres to the graphene surface, allowing low concentration to avoid protein aggregation.”

o Author should clarify whether the “5-fold excess folded tRNA” refers to molar excess and whether tRNA was pre-incubated under aminoacylation conditions.

We have clarified as follows to Materials and Methods/Cryo-EM sample preparation:

“4 μL of 0.04 mg/mL or 0.04 mg/mL MpMetRS with 5-fold molar excess folded tRNA^Met^, in the absence of the aminoacyl substrate”

2. Data Processing:

o The transition from symmetric (C2) to asymmetric refinement due to “ill-defined additional features” is not sufficiently justified. Provide quantitative metrics (e.g., FSC curves, particle distribution) to support this decision.

We have clarified as follows to paragraph 2, Results/Cryo-EM data collection and processing:

Closer inspection of the 2D classes revealed some classes with blurred features that did not follow the 2-fold symmetry that dominated the initial classification (S1 Fig). Further, the GSFSC curve of the C2 dimer showed a bump at moderate resolution, indicative of non-resolved features in the map (S2 Fig) To explore the possibility that this density corresponded to the missing MetRS catalytic domain, this limited subset of particles (60,000) was used to refine a non-uniform, asymmetric map to 3.66 Å resolution. All maps were sharpened with deepEMhancer for enhanced visualization [19].

o The absence of density for the AARS catalytic domain is a major limitation. Discuss whether this is due to flexibility, partial occupancy, or resolution limits. Include a supplemental figure showing local resolution maps.

Unfortunately, this is not an uncommon limitation for structural analysis of AARS enzymes, which bind their substrates fairly weakly and by an induced fit mechanism. We have added the following details to new paragraph 5, Introduction:

“Many AARSs exhibit induced fit, both to select their cognate tRNAs based on nucleotide identities in the three-nucleotide anticodon and the acceptor stem and to discriminate amongst similar amino acid functional groups. Nevertheless, high-resolution structure determination techniques rely on specimen homogeneity, so many of the existing AARS structures have missing features that do not fully define the structural basis for the AARS mechanism. For example, tRNAs that are aminoacylated by class I sub-family AARSs necessarily undergo a “hairpin” rearrangement of the 3’-terminal acceptor end to approach the activated amino acid in the AARS catalytic site. Few high-resolution structures of such hairpinned tRNAs bound to their cognate AARSs are available. The modular nature of AARSs contributes to efficient catalysis and a wide array of novel functions but a complete model of the structural basis for the biochemical data remains elusive.”

3. Model Validation:

o Provide detailed validation statistics (CC, Ramachandran outliers, clash scores) for the deposited model (9QS7).

o The use of AlphaFold2 to model a symmetric dimer is interesting but should be framed as speculative. Clearly distinguish between experimental observations and computational predictions.

We added the following qualifiers (**bold**) to paragraph 2, Results/Modelling:

“To better understand how this steric clash **could** resolve, we compared the **experimental** results, which show an asymmetric and incompletely ordered dimer, with those from AlphaFold2 [35], imposing C2 symmetry. To adopt the fully symmetric dimer **predicted** by AlphaFold2, the helical subdomain from the MetRS domain **would** need to rotate about 60^o^ using the AGAT active site as a pivot point reference (Fig 6D). With that rotation, the MetRS catalytic domain is removed from the space between the two NTDs, opening the tRNA binding site. The 3’ end of the tRNA sits about 46 Å away from the AGAT active site.”

iv. Results

1. Structural Findings:

o The description of the AGAT dimer interface is clear, but the analysis of conserved vs. unique interactions would benefit from a quantitative comparison (e.g., buried surface area, interaction energies).

We added information about the buried surface area of the conserved AGAT dimer from the dimer and the tetramer that forms at higher protein concentration:

Paragraph 2, Results/The central domain is a dimeric AGAT

“In each homolog, the dimer interface buries about 3504 Å^2^ (of a total of 23,060 Å^2^ for each monomer) and is mediated by hydrophobic stacking interactions, salt bridges, and hydrogen bonds (Fig 3B).”

Paragraph 1, Results/ MpMetRS oligomeric state depends on the protein concentration

“Within those higher-order oligomers, the NTD and AGAT domains appear concatenated, with the NTD domain of one polypeptide positioned in opposition to the AGAT domain of the next polypeptide to bury an additional 1575 Å^2^.”

Paragraph 5, Discussion:

“In fact, the asymmetric unit of the TdPntC-AEPT also contains a dimer of dimers that buries 1154 Å^2^ [26], but where the second dimer is on a different face of the primary PntC-AEPT dimer from where it is in MpMetRS (Fig 7B).”

o The claim that the N-terminal domain is a nucleotidyl transferase is based on DALI results but lacks experimental validation (e.g., activity assays). This should be toned down or explicitly noted as a prediction.

We have changed our nomenclature for the N-terminal domain of unknown function to “NTD” throughout (outlined in Table 1).

**Table 1: Nomenclature**

| **Abbreviation** | **Usage** |
| --- | --- |
| *metS* | gene encoding *Mycoplasma penetrans* MetRS |
| MpMetRS | full-length *M. penetrans metS* gene product |
| MetRS | general MetRS domain |
| AGAT | alanine-glyoxylate aminotransferase domain |
| NTD | N-terminal domain |
| AARS | general aminoacyl-tRNA synthetase |
| NTase | general nucleotidyl transferase |

2. Asymmetry and Mobility:

o The observation that only the N-terminal and AGAT domains follow C2 symmetry is intriguing. However, the manuscript does not sufficiently explore the functional implications of this asymmetry. Is the asymmetry fixed or dynamic?

Given that we do not see density for the second MetRS domain, we presume it is from a dynamic protein but these distinctions are difficult to assess from a static technique like single particle cryo-EM. We address this by summarizing Budiman, *et al* (reference 37), prior molecular dynamics that show the N-terminal domain of *E. coli* MetRS is highly dynamic in paragraph 3, Discussion:

“Prior molecular dynamics simulations performed on a monomeric form of EcMetRS, which shares 25% sequence identity and 45% sequence similarity with the MetRS domain of MpMetRS, predicted that a zinc-binding insertion between halves of the active site, a surface loop that caps the active site, and the anticodon binding domain are particularly mobile and implicated in MetRS function [37]. This implied role of dynamics in tRNA aminoacylation also likely explains why these elements are not visualized in the averaged cryo-EM structure of MpMetRS even in the presence of added Met-tRNA.”

o The missing density for one AARS domain is a critical point. Consider whether this could be due to proteolytic cleavage or flexible linkage. SDS-PAGE (Fig S2) should be referenced here to confirm full-length protein integrity.

We clarify this as follows in paragraph 1, Results/The position of the MetRS domain does not follow C2 symmetry

“There is no well-resolved density for the N-terminal catalytic site of MetRS or for MetRS from the opposing subunit, despite PAGE-analysis showing the purified full-length protein that is predominantly a dimer (S6A Fig).”

3. Oligomerization at High Concentration:

o The reported tetramer/hexamer formation is interesting but preliminary. Provide size-exclusion chromatography or analytical ultracentrifugation data to corroborate the oligomeric state in solution.

We added these results in Supplemental Figure 6.

4. Figures:

o Figures are generally informative but could be improved:

§ Fig 3: Label the domains consistently (NTase, AGAT, AARS).

We have endeavored to use consistent nomenclature throughout, as outlined in Table 1 (above).

§ Fig 6: The docking models are useful but should include a measure of uncertainty (e.g., RMSD from multiple docking runs).

We added figures for the superimposition of the core elements for the DALI results in Tables 2 and 3, as well as visually in new Figures 2A and 3A. For the docking of the AARS domains, we included the following information in paragraph 1, Results/Modelling:

“Docking the helical bundle from monomeric *E. coli* MetRS (PDBID 1QQT) [31] predicts that the catalytic domain would fall immediately between the C2-symmetric NTDs (Fig 6B) (R.M.S.D of 1.0 Å for a core of 108 residues). In contrast, docking the tRNA-bound *E. coli* CysRS [32] onto the helical bundle of MpMetRS shows that the tRNA would sterically clash with the N-terminal dimer as positioned in the apo structure (Fig 6C) (R.M.S.D of 1.2 Å for a core of 40 residues).”

Paragraph 2, Results/Modelling

“With that rotation, the MetRS catalytic domain is removed from the space between the two NTDs, opening the tRNA binding site (R.M.S.D of 1.3 for a core of 111 residues between the MetRS domain and EcCysRS).”

v. Discussion

The discussion is somewhat speculative and should be more tightly linked to the structural observations.

1. Functional Interpretation:

o The proposal that the NTase domain “increases local ATP concentration” is interesting but untested. Suggest future experiments (e.g., ATPase activity assays) or temper the claim.

We removed this from the Discussion.

o The idea of substrate channeling between AGAT and MetRS domains is appealing but not supported by direct evidence. A more cautious interpretation is warranted.

We reframed this in the context of of the known capability for the MetRS domain to use Met synthesized by the AGAT domain (Murowski, et al, reference 3) in the revised Discussion.

2. Evolutionary Context:

o Expand on why such a complex fusion evolved in a genome-reduced organism. Could this be a compensatory mechanism for host dependence?

We do not feel our structure can inform this intriguing suggestion.

3. Limitations:

o The resolution (3.66 Å) is moderate for detailed mechanistic insights, especially for flexible regions. Acknowledge this limitation.

We have done so as follows in paragraph 1, Results/The position of the MetRS domain does not follow C2 symmetry.

“The resulting structure shows additional density with helical features, albeit at limited resolution (3.66 Å resolution) (Fig 4B).”

o The lack of a tRNA-bound high-resolution structure limits functional conclusion. Mention this as a key area for future work.

We have acknowledged this in paragraph 2, Results//The position of the MetRS domain does not follow C2 symmetry.

“Identifying conditions where the tRNA binds for structural analysis remains an active area of exploration.”

vi. Data Availability

The deposition of coordinates (9QS7) and maps (EMD-70794) is commendable. Ensure that the half-maps and processing metadata are also publicly accessible to allow independent validation.

Half maps were submitted to the EMDB and will be available when the coordinates are released.

vii. Overall Assessment

This study presents a novel and interesting structure of a chimeric MetRS with potential implications for understanding domain fusion and enzyme adaptation in minimal genomes. The cryo-EM work is technically sound, but the manuscript currently overinterprets the data in several places and lacks critical validations.

We endeavored to qualify and clarify the possible significance of this unique protein fusion in an organism with an otherwise minimal genome, for example in Conclusion:

“First, **if** the NTD serves as a NTase, it would increase the availability of the precious cellular resource ATP. Second, the AGAT domain synthesizes methionine to **possibly** supply the aminoacylation catalytic domain. Finally, the mobile MetRS domain **could** position its catalytic site to receive these essential metabolites before binding tRNA^Met^. **Future work will evaluate the synergy that benefits *M. penetrans* from fusing these different enzyme activities.**

Major revisions are required to:

1. Clarify methodological details, especially regarding sample preparation and data processing choices.

2. Temper functional claims that are not experimentally supported.

3. Strengthen the discussion by linking structural observations more directly to possible biological mechanisms.

4. Improve the presentation of figures and models to better convey asymmetry and conformational flexibility.

We endeavored to address each of these points, as outlined above.

With these revisions, the manuscript could make a valuable contribution to the fields of structural biology and molecular evolution.

Thank you for your helpful and constructive suggestions.

Recommendation: Major Revision

Sincerely,
Dr. Morufu Olalekan RAIMI, BSc, (Geography. and Environmental Management), Diploma. (Environmental Health), M.Sc. Environmental Health Management), M.Phil. (Environmental Health Science), P.hD (Environmental Health Science), MNES, REHO, LEHO, FAIWMES
Environmental Health Consultant/Lecturer at Federal University Otuoke, Bayelsa State. Nigeria.
Environmental Health Consultant to United Nations Economic Commission for Europe (UNECE) Expert Group on Resources Management (EGRM). Geneva, Switzerland.

Research Consultant to Bayelsa State Primary Health Care Board.

Former Technical Adviser to the Executive Secretary, Bayelsa State Primary Health Care Board.
Former Director, Advocacy, Communication and Social Mobilization, Bayelsa State Primary Health Care Board.

Program Manager, Centre for Niger Delta Studies and Sustainability (CNDSS), Federal University Otuoke, Bayelsa State.

Deputy Director, Niger Delta Institute for Emerging and Re-emerging Infectious Diseases (NDIERID), Federal University Otuoke, Bayelsa State.

Reviewer to National Science Foundation (NSF) Graduate Research Fellowship Program (GRFP)
Plos One Academic Editor

https://publons.com/a/1479339/
https://ssrn.com/author=2891311
ORCID iD: <https://orcid.org/0000-0001-5042-6729>

Web of Science Researcher ID: <https://publons.com/a/1479339>

Website: https://ssrn.com/author=2891311; https://www.growkudos.com/profile/morufu_raimi; https://sciprofiles.com/profile/Morufuolalekanraimi; <https://livedna.org/234.27529>

https://scholar.google.com/citations?user=nRBW82AAAAAJ&hl=en.
https://theconversation.com/profiles/morufu-olalekan-raimi-1520774

**Reviewer #3:**

The manuscript by B.G. Esfahani and co-authors investigates the structure of the methionyl-tRNA synthetase (MetRS) from the pathogen Mycoplasma penetrans (Mp). Despite the highly reduced genome of this bacterium, MpMetRS is an unusually large protein (1087 amino acids, 126.4 kDa), owing to an additional module appended to the N-terminus of the canonical MetRS enzyme. Previous biochemical studies have shown that full-length MpMetRS is expressed in vivo, with its N-terminal module mediating dimerization in vitro and containing an alanine-glyoxylate aminotransferase (AGAT) domain involved in methionine biosynthesis. Notably, the methionine produced by the AGAT domain can be used by the MetRS domain to synthesize Met-tRNAMet in vitro, suggesting potential substrate channeling and an intriguing evolutionary adaptation.

In the present study, authors determined the structure of MpMetRS at 3.66Å resolution using single-particle cryo-electron microscopy (cryo-EM), revealing a three-domain architecture comprising an N-terminal nucleotidyl transferase (NTase) domain (residues 1 to 166), followed by the AGAT domain (residues 167 to 560), and the C-terminal MetRS domain (residues 561 to 1087). The identification of the N-terminal NTase domain represents a major contribution of this work, providing a more complete view of the modular organization of MpMetRS. The cryo-EM structure is consistent with the previously reported dimeric nature of MpMetRS and confirms that dimerization is driven by the AGAT domain, whose mode of association closely resembles that observed in related AGAT proteins (e.g. Anabaena), while also displaying interactions specific to M. penetrans. The NTase and AGAT domain adopt a well-defined two-fold symmetric arrangement, whereas the MetRS domain is only partially resolved and appears asymmetrically positioned. Despite extensive attempts to visualize tRNA-bound states, no well-defined density corresponding to bound tRNA or to fully resolved MetRS domain could be obtained, indicating significant conformational flexibility of this region. Based on docking of orthologous MetRS-tRNA complexes and AlphaFold2 modeling, the authors propose that significant domain arrangements would be required to accommodate tRNA. Finally, at higher protein concentration and using a different strategy for grid preparation, the authors observed higher-order oligomeric assemblies involving the NTase and AGAT domains, although their biological relevance remains unclear. Overall, the study provides structural insights into the organization, symmetry, and oligomeric state of this multidomain MetRS enzyme, while emphasizing the dynamic nature of its catalytic domain.

Although this work is of undeniable interest, some concerns must be addressed to improve the presentation and interpretation of results.

Major concerns:

1) The main strength of this study is the resolution of the structure of the N-terminal module of MpMetRS, which consists of an NTase domain followed by an AGAT domain. The identification of the NTase domain is a particularly important contribution, as this domain had not been annotated on the modular description of the enzyme. However, a description of the overall architecture of the NTase domain is missing from the Results section and is only briefly mentioned in the Discussion section. Similarly, although the dimerization interface of the MpMetRS AGAT domain is described and compared with Anabaena AGAT dimer, a description of the overall MpMetRS AGAT structure and the “canonical” dimerization mode of this class of enzymes would improve the clarity and completeness of this section.

Thank you for the excellent suggestion. We have endeavored to enhance our discussion of the positive identification of the N-terminal domain (NTD) as a Rossman fold-containing nucleotidyl transferase (NTase) via a structure-based homology search (new Figures 2 and S3). We enhanced our comparison of the alanine glyoxylate aminotransferase (AGAT) domain with other homologs, in particular another example of a fusion between an NTase and AGAT from *Treponema denticola* (PDBID 6PD1, Rice, *et al*, reference 26) (new figures 3 and S4).

2) The successful application of single-particle cryo-EM to a large, flexible, and multidomain enzyme such as MpMetRS is challenging. While the NTase and AGAT domains were fully resolved, only the C-terminal anticodon-binding domain of one of the two MpMetRS protomers could be modeled based on experimental data. The N-terminal module of MpMetRS, comprising the NTase and AGAT domains, dimerizes through the AGAT domain, resulting in a stable dimeric core with C2 symmetry. However, it is unclear whether the asymmetrical position of the observed C-terminal MetRS domain reflects true structural asymmetry of the MpMetRS enzyme or simply flexibility. Additional discussion or evidence is needed to clarify this point. The title of the study might be reconsidered.

We agree that this is a difficult distinction to make using a static technique like single particle cryo-EM. We endeavored to address this issue by referencing prior molecular dynamics of *E. coli* MetRS (Budiman*, et al*, reference 37) as described in response to Reviewer 2’s comments.

We have re-titled the manuscript “*Mycoplasma penetrans* methionyl-tRNA synthetase dimerizes via tandem N-terminal ancillary domains”.

3) The observation of higher-order oligomeric assemblies at elevated protein concentration and under specific grid preparation conditions is fascinating, but their biological relevance remains to be investigated. It is unclear whether these oligomers exist in solution under native conditions or arise as an artifact of cryo-EM grid preparation, and the authors should clarify this point. In the absence of additional experiments (e.g. size-exclusion chromatography, dynamic light scattering) supporting concentration-dependent oligomerization of NTase-AGAT units, the potential functional role of these oligomers should be more clearly emphasized as speculative. I suggest presenting these observations after the “Modeling” section in Results and condensate Figures 5 and 7 into one.

We have reorganized the figures as suggested and added context to the concatenation by comparing it to the crystal structure of the PntC-AEPT from *T. denticola* while stating the following

“We further observed a concentration-dependent effect on the oligomeric state of MpMetRS. Oligomerization of nucleotide and polynucleotide binding proteins is not atypical, for example in the DNA recombination protein RecA [38], cytidine triphosphate synthase [39] or the G-quadruplex binding protein nucleoside diphosphate kinase [40] but the *in vivo* significance of this observation for MpMetRS remains to be determined. “

4) Despite extensive efforts, no clear density was observed for the MetRS domain in complex with tRNA. Nevertheless, the authors discuss steric constraints and domain rearrangements required for tRNA binding based on docking with orthologous MetRS-tRNA complexes and on AlphaFold2 predictions. While these approaches are reasonable, they cannot substitute for experimental validation. Techniques such as small-angle X-ray scattering (SAXS) could provide evidence for the large conformational changes associated with tRNA binding and would strengthen the study. In the absence of such additional data, authors should more explicitly acknowledge the limitations of the study and further emphasize the speculative nature of the proposed mechanisms for tRNA binding. In addition, I suggest including and discussing AlphaFold3 models of MpMetRS alone and in complex with tRNAMet, which might provide further insights into these potential structural rearrangements.

This is a good suggestion, however we experienced an error when we attempted to model the complete structure in AlphaFold3.

5) The authors propose potential functional coupling between NTase, AGAT, and MetRS domains as an evolutionary advantage of this multidomain MetRS enzyme. While this hypothesis is interesting and appropriately presented as speculative, the concluding paragraph does not sufficiently emphasize the main contribution of the study that is directly supported by experimental data: the structure of the M. penetrans-specific N-terminal module of MetRS, comprising an NTase and AGAT domain that drives dimerization. The authors should refocus the conclusion and integrate the hypothesis of functional coupling withing the Discussion section.

We endeavored to streamline and clarify the discussion and conclusions, as outlined in our response to Reviewer 2.

Minor concerns:

1) A sentence summarizing the results of the study by Jones. T.E. et al. (2008) (doi : 10.1016/j.molcel.2007.12.021) should be included in the introduction. It would be useful to state that the N-terminal module of MpMetRS is not involved in the unusual mechanism of tRNA substrate discrimination described in that study.

We have added the following statement to paragraph 4, Introduction:

“The isolated MetRS domain is able to selectively aminoacylate its cognate tRNA^Met^ and reject the near-cognate tRNA^Ile2^ by contacting acceptor stem nucleotides remote from the tRNA anticodon [8].”

(Jones, *et al*, is reference 8).

2) The sentence at line 77 of the Introduction appears redundant, as the preceding sentence already states that the N-terminal-most domain corresponds to an NTase domain.

3) In the sentence at line 78 of the Introduction, the authors should explicitly indicate which enzymes domains are organized around the AGAT domain.

In response to comments 2 and 3, we edited the introduction to remove redundancy and clarify our nomenclature, Table 1 (above) and paragraph 6, introduction).

Here, we report the 3.66 Å-resolution structure of MpMetRS that reveals a chimera of three structurally independent protein modules: 1) a previously uncharacterized NTD, 2) a dimeric AGAT domain, and 3) a single helical bundle from one of the two MetRS domains. Structural homology of the NTD most closely aligns with a large class of nucleotidyl transferases (NTases). This NTD and the central AARS domain organize around the AGAT-dominated two-fold symmetry axis independently such that only the N-terminal and AGAT domains follow C2 symmetry. This is the first example, to our knowledge, of this collection of domains in a MetRS, allowing speculation that the metabolic functions may be coupled in this organism with an otherwise minimal genome.

4) In the sentence at line 84 of the Material and Methods section, the authors should add the GeneBank identifier of the metS gene and justify the use of the M568A MpMetRS mutant for the structural analysis.

We have done this in paragraph 1, Material and Methods/Preparation of Biological Specimen:

“Full length *metS* (GenBank MYPE9380) harboring a mutated codon for the M568A alteration to remove an internal start codon was cloned into pQE70 behind an N-terminal six-histidine tag [3, 8].”

5) Showing the elution profile of MpMetRS on the Sephacryl S300 size-exclusion column as a supplementary figure might provide additional evidence for conformational heterogeneity.

We added SEC and DLS analysis in Supplemental Figure 6.

6) Including additional AGAT sequences, modeled by AlphaFold2 or AlphaFold3, in the structure-based alignment shown in Figure S1 could help better identify interactions that are conserved among AGAT dimers and those that are specific to Mycoplasma.

We added DALI structural analysis for AGAT and enhanced our discussion of the conserved dimer/tetramer interface as new Figure 3 and Supplemental Figure 4.

7) In the legend of Figure 1, the inset is not described.

We have revised and updated all figures and legends.

8) In the legend of Figure 3, the MetRS domain displayed in cartoon representation is not mentioned. The name of the different ligands shown in spheres should be also mentioned.

We have revised and updated all figures and legends.

9) A scheme representing the modular organization of MpMetRS would strengthen the clarity of the manuscript. This might be included as part of Figure 1.

We have added this scheme as new Figure 1A.

10) Several figures in the Results section are difficult to interpret (e.g. Figures 1, 4, and 5). Their clarity could be improved by using color coding for the different domains, indicating symmetry axes, and including schematic diagrams that illustrate the overall domain organization (as shown in Figure 5B).

We have revised and updated all figures and legends.

11) Figure 7 and its legend need to be improved by showing also the cartoon representation of the concatenated NTase-AGAT subunits. The nature of the ligands in the active sites should be stated in the legend. The region zoomed should be described.

We have revised and updated all figures and legends.

12) Provide supplementary tables summarizing cryo-electron microscopy data collection parameters and refinement statistics.

We have added this as new Supplemental Figure 1 and Supplemental Table 1.

Reviewer #4: The contribution entitled Mycoplasma penetrans methionyl tRNA synthetase is an asymmetric dimer fused to N-terminal ancillary domains by Esfahani et al., reports a partial structure of the unusual Mycoplasma penetrans methionyl-tRNA synthetase (MpMetRS) at a 3.7 A resolution. The MpMetRS has been previously shown to contain an aminotransferase domain fusion which is able to catalyze conversion of 2-keto-4-methylthiobutyrate (KMTB) to methionine and that this methionine can be subsequently transferred to tRNAMet in vitro (Muraski et al., 2020). Here, the authors were able to solve the structure of the aminotransferase domain along with the N-terminal nucleotidyl transferase domain. The topic is highly interesting, particularly the functional roles of fusion enzymes in minimal-genome organisms and the enrichment of enzymatic capabilities through the acquisition of novel domains. However, the manuscript is difficult to follow, as key background information is missing and the conclusions are not always fully supported by the presented results.

Major comments:

Abstract: From the abstract, it is not clear which findings are directly supported by the data and which are speculative. In addition, the simultaneous use of the full-length MetRS and the isolated MetRS domain is somewhat confusing.

We have endeavored to clarify the Abstract and nomenclature, outlined in new Table 1 (above).

Abstract:
“Diverse aminoacyl-tRNA synthetase (AARS) gene fusions are now recognized as a common mechanism for enhancing genetic diversity across all domains of life. The *metS* gene from *Mycoplasma penetrans* is a striking example of such an evolutionary mechanism because although *M. penetrans* has a condensed genome, the *metS* gene is nearly twice the size of a typical bacterial gene encoding methionyl-tRNA synthetase (MetRS). We used cryo-EM to analyze the structure of the MpMetRS gene product to show that it is the product of three distinct enzyme domains: an N-terminal domain (NTD) of unknown function, a dimeric alanine-glyoxylate aminotransferase, and a MetRS. Only the first two N-terminal domains show two-fold symmetry, which were resolved to 3.27 Å resolution and the MetRS domain is only partially resolved to 3.66 Å resolution. Modelling the full structure shows that a conformational change must occur to accommodate a tRNA-bound MetRS domain. Further rearrangement of the catalytic domains would also be necessary to bring the active sites adjacent to one another if this unique assembly of catalytic domains functions to channel substrates to MetRS.”

Introduction: The background information on MetRS is in my opinion insufficient. For example, the manuscript does not clearly describe the canonical architecture of MetRS enzymes, or identify which known MetRS enzyme is the MpMetRS most closely related to. It is unclear whether it is expected to function as a monomer or dimer. It is also unclear whether the activity of the N-terminal NTase domain has been previously established. A schematic domain diagram of MpMetRS would greatly aid clarity, as the current text makes it difficult to distinguish actual architecture of the MpMetRS (e.g., it is sometimes difficult to understand whether it is the N-terminal NTase domain or the N-terminal region of the MetRS (“MetRS domain”)). In addition, the final sentence of the Introduction is not very clear and would benefit from further elaboration.

We have added to the information in the Introduction (for example new paragraph 4) and added Figure 1A and Table 1 to help with clarity.

Results: Overall, it remains difficult to discern what is definitively demonstrated in the manuscript. For example, the assignment of the “additional density with helical features” to the C-terminal anticodon-binding domain is not sufficiently justified, and the level of confidence in this interpretation is unclear. Similarly, the rationale for modeling the tRNA using E. coli CysRS:tRNACys rather than the MetRS:tRNAMet structure (Nakanishi et al., 2005) is not explained.

We added information about the structural similarity of the helical domain with the conserved *E. coli* MetRS structure (RMSD of 1 Å). We added the following rationale for using the *E. coli* CysRS-tRNA_cys_ structure for comparison:

“The tRNA-bound CysRS co-crystal structure (PDBID 1U0B [32]) was used as a template because it captures the hairpinned tRNA acceptor stem directed into the AARS active site; a cocrystal structure of *A. aeolicus* MetRS:tRNA^Met^ is available (PDBID 2CSX [33]), however in this structure the tRNA 3’-end is disordered and the acceptor stem does not approach the active site.”

Several figure legends are incomplete, and Figure 6 in particular is difficult to interpret, as the aligned and clashing regions are not clearly presented. The conclusion that MpMetRS forms a dimer (line 210) is also not well supported, given that the native gel (Fig. S2) suggests species closer to trimers or tetramers and that tetrameric assemblies are observed in cryo-EM at higher concentrations.

We endeavored to clarify the oligomeric state of the complex by including SEC and DLS data (new Figure S6), which is indeed concentration dependent. We clarify that further experimentation would be needed to determine its oligomeric state in the cell.

“We further observed a concentration-dependent effect on the oligomeric state of MpMetRS. Oligomerization of nucleotide and polynucleotide binding proteins is not atypical, for example in the DNA recombination protein RecA [38], cytidine triphosphate synthase [39] or the G-quadruplex binding protein nucleoside diphosphate kinase [40] but the *in vivo* significance of this observation for MpMetRS remains to be determined.”

In addition, it is unclear why tRNA-containing samples were not examined using the chameleon plunging system, or whether attempts were made to isolate a defined MpMetRS:tRNAMet complex prior to cryo-EM, rather than relying on excess tRNA during grid preparation.

We imaged/analyzed both Vitrobot and chameleon-prepared MpMetRS/tRNA complex but unfortunately the results were the same. We attempted to co-purify the complex but the tRNA did not remain bound over SEC.

We clarified this in Methods/Cryo-EM sample preparation

“MpMetRS (4 μL of 0.04 mg/mL or 0.04 mg/mL MpMetRS with 5-fold molar excess folded tRNA^Met^, in the absence of the aminoacyl substrate, was applied to a hydrophilized graphene-coated [12] holey carbon-on-gold Quantifoil cryo-EM grid (Quantifoil, Jena, Germany). MpMetRS adheres to the graphene surface, allowing low concentration to avoid protein aggregation. Plunging into liquified ethane after a blot force of 4, 1s blot was performed on a Vitrobot Mark IV (Thermo Scientific), resulting in thin ice and an even distribution of monodispersed particles. Alternatively, 10 mg/mL MpMetRS or MpMetRS with 5-fold molar excess tRNA^Met^ was applied to a nanowire self-wicking grid using the chameleon® (SPT Labtech, Melbourne, UK) nanospray device [13] with a 54 ms wicking time.”

6. PLOS authors have the option to publish the peer review history of their article ([what does this mean?](https://journals.plos.org/plosone/s/editorial-and-peer-review-process#loc-peer-review-history)). If published, this will include your full peer review and any attached files.


If you choose “no”, your identity will remain anonymous but your review may still be made public.


**Do you want your identity to be public for this peer review?** For information about this choice, including consent withdrawal, please see our [Privacy Policy](https://www.plos.org/privacy-policy).

Reviewer #1: No

Reviewer #2: **Yes:** Morufu Olalekan Raimi Ph.D

Reviewer #3: No

Reviewer #4: No

[NOTE: If reviewer comments were submitted as an attachment file, they will be attached to this email and accessible via the submission site. Please log into your account, locate the manuscript record, and check for the action link "View Attachments". If this link does not appear, there are no attachment files.]

To ensure your figures meet our technical requirements, please review our figure guidelines: https://journals.plos.org/plosone/s/figures

You may also use PLOS’s free figure tool, NAAS, to help you prepare publication quality figures: https://journals.plos.org/plosone/s/figures#loc-tools-for-figure-preparation.

NAAS will assess whether your figures meet our technical requirements by comparing each figure against our figure specifications.
