# Supplementary material for: Mycoplasma penetrans methionyl-tRNA synthetase dimerizes via tandem N-terminal ancillary domains
Source: PLoS One. 2026 May 27;21(5):e0347747. doi: 10.1371/journal.pone.0347747 (PMC13215547; doi:10.1371/journal.pone.0347747)
Supplement: Attachment — Submitted filename: PONE-RtR2_033026.docx [file pone.0347747.s004.docx]

Please include the following items when submitting your revised manuscript:

- A letter that responds to each point raised by the academic editor and reviewer(s). You should upload this letter as a separate file labeled 'Response to Reviewers'.
- A marked-up copy of your manuscript that highlights changes made to the original version. You should upload this as a separate file labeled 'Revised Manuscript with Track Changes'.
- An unmarked version of your revised paper without tracked changes. You should upload this as a separate file labeled 'Manuscript'.

If you would like to make changes to your financial disclosure, please include your updated statement in your cover letter. Guidelines for resubmitting your figure files are available below the reviewer comments at the end of this letter.

Journal Requirements:

1. If the reviewer comments include a recommendation to cite specific previously published works, please review and evaluate these publications to determine whether they are relevant and should be cited. There is no requirement to cite these works unless the editor has indicated otherwise.

2. Please review your reference list to ensure that it is complete and correct. If you have cited papers that have been retracted, please include the rationale for doing so in the manuscript text, or remove these references and replace them with relevant current references. Any changes to the reference list should be mentioned in the rebuttal letter that accompanies your revised manuscript. If you need to cite a retracted article, indicate the article’s retracted status in the References list and also include a citation and full reference for the retraction notice.


[Note: HTML markup is below. Please do not edit.]

Reviewers' comments:

Reviewer's Responses to Questions

**Comments to the Author**

1. If the authors have adequately addressed your comments raised in a previous round of review and you feel that this manuscript is now acceptable for publication, you may indicate that here to bypass the “Comments to the Author” section, enter your conflict of interest statement in the “Confidential to Editor” section, and submit your "Accept" recommendation.

Reviewer #2: All comments have been addressed

Reviewer #3: All comments have been addressed

Reviewer #4: All comments have been addressed

2. Is the manuscript technically sound, and do the data support the conclusions?

The manuscript must describe a technically sound piece of scientific research with data that supports the conclusions. Experiments must have been conducted rigorously, with appropriate controls, replication, and sample sizes. The conclusions must be drawn appropriately based on the data presented.

Reviewer #2: Partly

Reviewer #3: Yes

Reviewer #4: Yes

3. Has the statistical analysis been performed appropriately and rigorously?

Reviewer #2: Yes

Reviewer #3: Yes

Reviewer #4: Yes

4. Have the authors made all data underlying the findings in their manuscript fully available?

The [PLOS Data policy](https://nam04.safelinks.protection.outlook.com/?url=http%3A%2F%2Ftrack.editorialmanager.com%2FCL0%2Fhttp%3A%252F%252Fwww.plosone.org%252Fstatic%252Fpolicies.action%2523sharing%2F1%2F010f019d18cfe5a9-79e76fbd-36b4-45af-a5f7-1e1183f0ca70-000000%2Fle0f5cigWvYugbJV4_8frEs5GW9MLbdLlGY078NW1tw%3D255&data=05%7C02%7Cmestroupe%40bio.fsu.edu%7C8ea973a5ca5d4c617f9708de888f31ef%7Ca36450ebdb0642a78d1b026719f701e3%7C0%7C0%7C639098345765673605%7CUnknown%7CTWFpbGZsb3d8eyJFbXB0eU1hcGkiOnRydWUsIlYiOiIwLjAuMDAwMCIsIlAiOiJXaW4zMiIsIkFOIjoiTWFpbCIsIldUIjoyfQ%3D%3D%7C0%7C%7C%7C&sdata=D4f8ccQYzmk5QzAxoA%2BNzX0u5WpQMi9zNXRg1Bgj2YU%3D&reserved=0) requires authors to make all data underlying the findings described in their manuscript fully available without restriction, with rare exception (please refer to the Data Availability Statement in the manuscript PDF file). The data should be provided as part of the manuscript or its supporting information, or deposited to a public repository. For example, in addition to summary statistics, the data points behind means, medians and variance measures should be available. If there are restrictions on publicly sharing data—e.g. participant privacy or use of data from a third party—those must be specified.

Reviewer #2: Yes

Reviewer #3: Yes

Reviewer #4: Yes

5. Is the manuscript presented in an intelligible fashion and written in standard English?

PLOS ONE does not copyedit accepted manuscripts, so the language in submitted articles must be clear, correct, and unambiguous. Any typographical or grammatical errors should be corrected at revision, so please note any specific errors here.

Reviewer #2: Yes

Reviewer #3: Yes

Reviewer #4: Yes

6. Review Comments to the Author

Please use the space provided to explain your answers to the questions above. You may also include additional comments for the author, including concerns about dual publication, research ethics, or publication ethics. (Please upload your review as an attachment if it exceeds 20,000 characters)

Reviewer #2: Manuscript Number: PONE-D-25-61422R1

Title: Mycoplasma penetrans methionyl-tRNA synthetase dimerizes via tandem N-terminal ancillary domains

Recommendation: Minor Revision

Review Date: 2026-03-13

Reviewer Comments

Dear Authors,

I. Title

The revised title accurately reflects the study's main finding.

• Current Title: “Mycoplasma penetrans methionyl-tRNA synthetase dimerizes via tandem N-terminal ancillary domains”

The title is clear, descriptive, and appropriately focused on the key structural insight—that dimerization is mediated by the N-terminal domains (NTD and AGAT). This is a significant improvement over the previous title, which emphasized asymmetry. The current title is more accurate given that asymmetry is inferred from partial density rather than directly observed for both domains.

• Recommendation: Retain the current title.

Thank you.

II. Abstract

The revised abstract is much improved and now clearly distinguishes experimental findings from modeling.
• Minor Issues:

1. Phrasing: “We used cryo-EM to analyze the structure of the metS gene product (MpMetRS) to show that it is the product of three distinct enzyme domains” – slightly redundant (“product of the gene product”). Consider: “We used cryo-EM to determine the structure of the MpMetRS protein, revealing that it comprises three distinct enzyme domains.”

Thank you for the suggestion. The sentence now reads (bold indicates alteration throughout the Response to Reviewers):

“We used cryo-EM to analyze the structure of the *metS* gene product (MpMetRS) to show that it is the **fusion** of three distinct enzyme domains: an N-terminal domain of unknown function, a dimeric alanine-glyoxylate aminotransferase (**AGAT**), and a MetRS.”

2. Specificity: The abstract mentions “Modelling the full structure shows that a conformational change must occur.” It might be helpful to briefly indicate what kind of change (e.g., “rotation of the MetRS domain relative to the AGAT core”).

• Recommendation: Minor refinements as suggested.

The sentence now reads (bold to indicate alteration):

“Modelling the full structure **suggests that a rotation of the MetRS domain relative to the AGAT domain** must occur to accommodate a tRNA-bound MetRS.”

III. Introduction

The introduction is now comprehensive and well-structured, with clear progression from biological context to specific aims.

• Minor Issues:

1. Citation formatting: Some references in the introduction (e.g., references 3, 8) are cited with “Muraski et al., 2020” and “Jones et al., 2008” – these appear correctly in the reference list, but ensure all are consistently formatted.

We have carefully checked our references.

2. Terminology consistency: The introduction uses both “NTD” and “NTase” for the N-terminal domain. Table 1 clarifies that NTD is the domain name and NTase is the predicted function. This is helpful, but the introduction could briefly note that “based on structural homology, we identify this domain as a nucleotidyl transferase (NTase) fold, though its specific substrate remains unknown.”

We clarified this sentence in the final paragraph as follows:

“Structural homology of the NTD **fold** most closely aligns with a large class of nucleotidyl transferases (NTases), **though its function and substrate specificity remain speculative.**”

3. Final paragraph: The final sentence (“This is the first example, to our knowledge, of this collection of domains in a MetRS, allowing speculation that the metabolic functions may be coupled...”) is appropriate. However, it could be slightly more specific: “allowing speculation that the three activities, nucleotide metabolism, methionine biosynthesis, and tRNA aminoacylation, may be functionally coupled in this minimal-genome organism.”

This sentence now reads:

“This is the first example, to our knowledge, of this collection of domains in a MetRS, allowing speculation that the **three activities, nucleotide metabolism, methionine biosynthesis, and tRNA aminoacylation, may be functionally coupled in this minimal-genome organism.”**

• Recommendation: Minor refinements as suggested.

IV. Materials and Methods

The methods section is thorough and well-organized. The additions in response to reviewers have addressed previous concerns.

• Minor Issues:

1. tRNA preparation: The description of tRNA preparation is adequate but could be slightly expanded: "tRNA was resuspended in TE buffer; concentration was determined by absorbance at 260 nm." It might be helpful to note the extinction coefficient used or the approximate yield.

We added the extinction coefficient (40 (μg/mL)^-1^ cm^-1^).

2. Cryo-EM sample preparation: The description of the two preparation methods (low concentration on graphene grids vs. high concentration with chameleon) is clear. However, the sentence "MpMetRS adheres to the graphene surface, allowing low concentration to avoid protein aggregation” could be moved earlier for clarity.

That paragraph now begins:

“**MpMetRS adheres to the graphene surface, allowing low concentration to avoid protein aggregation. Therefore,** MpMetRS (4 μL of 0.04 mg/mL or 0.04 mg/mL MpMetRS with 5-fold molar excess folded tRNA^Met^, in the absence of the aminoacyl substrate), was applied to a hydrophilized graphene-coated [12] holey carbon-on-gold Quantifoil cryo-EM grid (Quantifoil, Jena, Germany).”

3. Data processing: The description of the transition from symmetric to asymmetric refinement is now well-justified with reference to 2D classes and GSFSC curves (S1 and S2 Figs). Good.

4. Model building and refinement: The description of model building with ModelAngelo and refinement in PHENIX is appropriate. The DALI searches are well-described. However, it would be helpful to include a brief statement about model validation statistics (e.g., “Refinement statistics, including map-model correlations, Ramachandran statistics, and clash scores, are provided in Supplemental Table 1”).

We added a citation of Table S1 to the appropriate sentence:

“Model building was initiated with the ModelAngelo [20] model builder. Iterative real-space refinement in PHENIX [21], with manual fitting in Coot [22], was performed independently for the core dimer and the C-terminal domain helical bundle of the MetRS domain (**S1 Table**).”

5. Dynamic light scattering: The description is clear. Minor: “Data was analyzed” should be “Data were analyzed” (plural).

Grammer fixed.

• Recommendation: Minor refinements as suggested, particularly adding model validation statistics to the methods or referring to them in Supplemental Table 1.

V. Results

The results are clearly presented and well-organized. The new figures and tables enhance readability.
• 5.1. The NTD has structural homology to an NTase: This section is excellent. The DALI results (Table 2) convincingly demonstrate the structural relationship. Figure 2 is clear and well-annotated. The description of the conserved core and divergent loops is precise and informative. The observation about the closed loop potentially occluding the nucleotide-binding pocket is interesting and appropriately noted.

• Minor: The sentence “Regions that are non-conserved are gray” in the Figure 2 legend is clear. In the text, the authors might briefly speculate whether the closed loop conformation is functionally relevant or an artifact of the apo state.

We added the following speculation:

“In contrast, the same loop in MpMetRS is longer and is closed, sterically obscuring the pocket and clashing with a potential nucleotide binding site. After a turn, residues 29-31 also form a loop before the first peripheral α-helix that, in MpMetRS, packs against the **AGAT** domain (Fig 2C and S3 Fig). In the other homologs analyzed here, this loop is free and further from the nucleotide binding pocket. **In this way, access to the nucleotide binding pocket could be related to the position of the subsequent AGAT domain.**”

• 5.2. The central domain is a dimeric AGAT: This section is similarly strong. Table 3 provides a useful overview of structural homologs. Figure 3 is clear, and the description of the dimer interface (with buried surface area and specific interactions) is appropriately quantitative. The comparison with TdPntC-AEPT (PDBID 6PD1) is illuminating and well-illustrated in Figure 3C.

• Minor: The sentence “In each homolog, the dimer interface buries about 3504 Å² (of a total of 23,060 Å² for each monomer)” – the total surface area for each monomer seems high (23,060 Å²). Is this the correct value? A typical 60 kDa domain might have ~20,000 Å² total surface area, but the AGAT domain is ~44 kDa (residues 167-560). Please verify.

We revisited the calculations, and they are accurate. We clarified as follows:

“In each homolog, the dimer interface buries about 3504 Å^2^ (of a total of 23,060 Å^2^ for each monomer **including the NTD and AGAT**) and is mediated by hydrophobic stacking interactions, salt bridges, and hydrogen bonds (Fig 3B).”

• 5.3. The position of the MetRS domain does not follow C2 symmetry: This section presents the key finding of asymmetry. The description is clear, and the authors appropriately note the limitations (limited resolution, no density for the second MetRS domain). The reference to PAGE analysis confirming full-length protein (S6A Fig) is important to rule out proteolysis. The observation of new 2D classes with tRNA (S7 Fig) is intriguing, even if high-resolution features were not obtained.

• Minor: The sentence “There is no well-resolved density for the N-terminal catalytic site of MetRS or for MetRS from the opposing subunit” – it might be helpful to explicitly state that this means the visible helical bundle corresponds to only one of the two MetRS domains, and that it is the C-terminal anticodon-binding domain, not the catalytic domain.

This sentence now reads:

“There is no well-resolved density for the N-terminal catalytic site of MetRS or for MetRS from the opposing subunit, despite PAGE-analysis showing the purified full-length protein that is predominantly a dimer (S6A Fig), **therefore we interpret the visible helical domain as coming from only one of the subunits and not including the catalytic domain.**”

• 5.4. Modelling: This section appropriately uses docking and AlphaFold2 to explore conformational possibilities. The rationale for using EcCysRS (PDBID 1U0B) as a template because it captures the hairpinned tRNA acceptor stem is well-explained. The predicted steric clash and the proposed ~60° rotation are clearly described. The RMSD values provide appropriate uncertainty measures.

• Minor: The sentence “Docking the helical bundle from monomeric E. coli MetRS (PDBID 1QDT) [31] predicts that the catalytic domain would fall immediately between the C2-symmetric NTDs (Fig 5B) (R.M.S.D of 1.0 Å for a core of 108 residues).” – “R.M.S.D.” is typically written as “RMSD”.

Done.

Also, ensure figure callouts are consistent (Fig 5B, 5C, 5D – the text refers to Fig 6 in places; please verify).

Figure numbering is now correct.

• 5.5. MpMetRS oligomeric state depends on the protein concentration: This section presents the intriguing observation of higher-order oligomers at high concentration. The SEC and DLS data (S6 Fig) provide valuable solution-state context. The comparison with TdPntC-AEPT in Figure 7 is appropriate. The authors appropriately note that the in vivo significance remains to be determined.

• Minor: The sentence “Within those higher-order oligomers, the NTD and AGAT domains appear concatenated, with the NTD domain of one polypeptide positioned in opposition to the AGAT domain of the next polypeptide to bury an additional 1575 Å².” – “NTD domain” is redundant (NTD already means N-terminal domain). Use “NTD” alone.

Done.

• Recommendation: Minor refinements as suggested, including verification of the surface area value and consistent figure referencing.

VI. Discussion

The discussion is now more focused and appropriately cautious. The revisions have addressed previous concerns about overinterpretation.

• Minor Issues:

1. Paragraph 2 (lines ~75-80 in the discussion): “To further explore the possibility that the active sites could be coupled to increase the local concentration of Met for aminoacylating tRNA, we modeled the apo anticodon binding subdomain based on the crystal structure of EcCysRS [31] (Fig 6).” – This should reference the correct figure (likely Fig 5 based on the revised numbering).

Please verify all figure callouts.

Done.

2. Paragraph 3: “Prior molecular dynamic simulations performed on a monomeric form of EcMetRS... predicted that the zinc-binding insertion... are particularly mobile.” – This is a helpful reference. The authors might also note that the mobility of these regions explains why they are not visualized in the cryo-EM structure, which is an average.

We clarified the appropriate sentence as follows:

“This implied role of dynamics **of those domains** in tRNA aminoacylation also likely explains why these elements are not visualized in the averaged cryo-EM structure of MpMetRS.”

3. Paragraph 4: “When the same docking is performed using a tRNA-bound AARS, EcCysRS [32], a conflict arises from the tRNA binding pose in relationship to the cleft between the central, C2-symmetric NTD/AGAT domain.” – The phrase "in relationship to" is awkward. Consider “relative to.”

Done.

4. Paragraph 5: “We further observed a concentration-dependent effect on the oligomeric state of MpMetRS.” – The discussion of oligomerization is appropriate, but the authors might briefly note whether similar concentration-dependent oligomerization has been observed for other NTase or AGAT family members.

We have added the following to the appropriate sentence:

“We further observed a concentration-dependent effect on the oligomeric state of MpMetRS. Oligomerization of nucleotide and polynucleotide binding proteins is not atypical, for example in the DNA recombination protein RecA [38], cytidine triphosphate synthase [39] or the G-quadruplex binding protein nucleoside diphosphate kinase [40]. **The** *in vivo* significance of this observation for MpMetRS remains to be determined **as we do not know of any specific other examples of concentration-dependent NTases or AGAT enzymes in isolation except for that of PntC-AEPT, described below.”**

5. Paragraph 6 (comparison with TdPntC-AEPT): This comparison is valuable. The authors might consider whether the different oligomerization modes (NTD-AGAT concatenation in MpMetRS vs. different interface in TdPntC-AEPT) might relate to the different domain organizations (NTD-AGAT-MetRS vs. PntC-AEPT).

Indeed, this is likely species specific as the NTD of unknown function in the enzyme studied here is structurally conserved with the NTase PntC from *Treponema denticola*.

We added the following discussion:

“The first two N-terminal domains of MpMetRS define its oligomeric state, but **in a different way than in** TdPntC-AEPT. More examples need to be identified and analyzed to determine the rules that control assembly and/or biological function, **but the determinants are likely species specific**.”

6. Limitations: The discussion acknowledges the moderate resolution and lack of tRNA-bound structure. However, it could also explicitly note that the functional assignments (NTase activity) are based on structural homology and await biochemical validation. This is already implied but could be stated directly.

We added this to the last sentence:

“Future **enzymatic** studies will evaluate the synergy that benefits *M. penetrans* from fusing these different **enzymatic domains**.”

• Recommendation: Minor refinements as suggested, particularly ensuring consistent figure referencing and considering a brief note about the need for biochemical validation of the predicted NTase activity.

VII. Conclusion

The conclusion is concise and appropriately focused on the study's contributions.

• Minor Issues:

1. Speculative language: The conclusion states “First, if the NTD serves as a NTase, it would increase the availability of the precious cellular resource ATP." This is appropriately conditional (“if”). However, the conclusion could also briefly note the key experimentally supported finding: that the NTD and AGAT domains form a stable dimeric core that mediates oligomerization.

We added this to the second point as only the AGAT contributes to the dimer interface:

“Second, the AGAT domain **serves as the core to the MpMetRS dimer** and synthesizes methionine to possibly supply the aminoacylation catalytic domain.”

2. Future directions: The final sentence (“Future work will evaluate the synergy that benefits M. penetrans from fusing these different enzyme activities.”) is appropriate but somewhat vague. Consider adding a brief indication of what such future work might entail (e.g., "through enzymatic assays of the isolated domains and full-length protein, and structural studies of substrate-bound complexes").

We added this to the last sentence:

“Future **enzymatic** studies will evaluate the synergy that benefits *M. penetrans* from fusing these different **enzymatic domains**.”

• Recommendation: Minor refinements as suggested.

With these minor revisions, the manuscript will be suitable for publication in PLOS One. I commend the authors for their thorough and responsive revision process and look forward to seeing the final version.

Reviewer #3: All comments have been addressed. I would just like to point out a few minor details:

- In Methods section (page 6), "Superdex" should be corrected to "Superose".

Error corrected.

- Figures 5 and 6 are not labeled as described in the text.

Corrected.

- I was able to model MpMetRS in complex with tRNA-Met using AlphaFold3 without difficulty. Since authors have significantly improved the quality of the manuscript, I will not insist on including or discussing this model. However, I encourage the authors to explore such modeling approaches in future studies.

Thank you for the suggestion.

Reviewer #4: The authors have satisfactorily addressed all of my comments. The manuscript is now technically sound and significantly improved in clarity, with extensive and well-executed revisions. The data adequately support the conclusions, and the statistical analyses have been performed appropriately. The manuscript is clearly written, and the underlying data are accessible.

Thank you.
